# Supplementary material for: Kinetochore inactivation by expression of a repressive mRNA
Source: eLife. 2017 Sep 14;6:e27417. doi: 10.7554/eLife.27417 (PMC5655150; doi:10.7554/eLife.27417)
Supplement: Supplementary file 1. — (A) Detailed genotypes for the strains used in this study. (B) Primers used for strain construction in this study. (C) Plasmids used for strain construction in this study. (D) Primers used for quantitative PCR and northern blotting in this study. (E) smFISH oligonucleotide probes used in this study. The NDC80ORF (Q 670) probe set consists of a mixture of thirty 20-mer oligonucleotide probes that tile the common region shared between NDC80luti and NDC80ORF. Each individual probe is labeled with the Quasar 670 dye. The NDC80Long (CF 590) probe set consists of a mixture of twenty 20-mer oligonucleotide probes that tile the unique 5’ region of NDC80luti. Each individual probe is labeled with the CAL Fluor Red 590 dye. smFISH oligonucleotide probes used in this study. The NDC80Odd (CF 590) probe set consists of a mixture of twenty-seven 20-mer oligonucleotide probes that tile NDC80ORF. Each individual probe is labeled with the CAL Fluor Red 590 dye. The NDC80Even (Q 670) probe set consists of a mixture of twenty-seven 20-mer oligonucleotide probes that tile NDC80ORF. Each individual probe is labeled with the Quasar 670 dye. (F) Summarized smFISH results for this study. (G) Predicted peptide sequences for the putative AUG uORFs. [file elife-27417-supp1.docx]

**Supplemental file 1A. Detailed genotypes for the strains used in this study.**

| **Strain** | **Genotype** |
| --- | --- |
| SK1  wild-type | *ho::LYS2 lys2 ura3 leu2::hisG his3::hisG trp1::hisG* |
| UB494 | *MAT***a** *ndc80-1* |
| UB877 | *MAT***a** */MATalpha ura3::pGPD1-GAL4(848).ER::URA3/ura3::pGPD1-GAL4(848).ER::URA3 GAL-NDT80::TRP1/GAL-NDT80::TRP1 leu2::pURA3-TetR-GFP::LEU2 CENV::TetOx224::HIS3 Ndc80-3V5:KanMX pCUP-CLB3::KANMX* |
| UB880 | *MAT***a** */MATalpha ura3::pGPD1-GAL4(848).ER::URA3/ura3::pGPD1-GAL4(848).ER::URA3 GAL-NDT80::TRP1/GAL-NDT80::TRP1 leu2::pURA3-TetR-GFP::LEU2 CENV::TetOx224::HIS3 KanMX:pCUP-Ndc80-3V5:CNAT pCUP-CLB3::KANMX* |
| UB885 | *MAT***a** */MATalpha ura3::pGPD1-GAL4(848).ER::URA3/ura3::pGPD1-GAL4(848).ER::URA3 GAL-NDT80::TRP1/GAL-NDT80::TRP1 leu2::pURA3-TetR-GFP::LEU2 CENV::TetOx224::HIS3 Ndc80-3V5:KanMX pCUP-CLB3::KANMX HISMX:pCUP-Spc25* |
| UB980 | *MAT***a** */MATalpha GAL-NDT80::TRP1/GAL-NDT80::TRP1 ura3::pGPD1-GAL4(848).ER::URA3/ura3::pGPD1-GAL4(848).ER::URA3 leu2::pURA3-TetR-GFP::LEU2 CENV::TetOx224::HIS3 Ndc80-3V5:KanMX pCUP-CLB3::KANMX pCUP-SPC24::KANMX* |
| UB1051 | *MAT***a** */MATalpha GAL-NDT80::TRP1/GAL-NDT80::TRP1 ura3::pGPD1-GAL4(848).ER::URA3/ura3::pGPD1-GAL4(848).ER::URA3 Spc25-3V5:HisMX/Spc25-3V5:HisMX* |
| UB1217 | *MAT***a** *HISMX:pGAL-Ndc80-3V5:KanMX ura3::pGPD1-GAL4(848).ER::URA3*  ***pGAL integrated 536 bp upstream of NDC80 AUG*** |
| UB1240 | *MAT***a** *Ndc80-3V5:KanMX ura3::pGPD1-GAL4(848).ER::URA3* |
| UB1323 | *MAT***a** *KanMX:pGAL-Δ9AUG-5'UTR-Ndc80-3V5:CNAT ura3::pGPD1-GAL4(848).ER::URA3*  ***pGAL integrated 536 bp upstream of NDC80 AUG***  ***ATG-ATC mutation in 9 of the 9 potential upstream start codons within NDC80-5'UTR. The leader sequence contains 2 SNPs from S288C introduced by a gene block and a third mutation (T🡪C) 8 bp after the 6^th^ ATG.*** |
| UB1337 | *MAT***a** */MATalpha pCUP-IME1::NAT/pCUP-IME1::NAT pCUP-IME4::NAT/pCUP-IME4::NAT Ndc80-3V5:KanMX/Ndc80-3V5:KanMX* |
| UB2388 | *MAT***a** *amn1::KanMX6 ura3::pGPD1-GAL4(848).ER::URA3 HISMX:pGAL-Ndc80-3V5:KanMX* ***pGAL integrated 536 bp upstream of NDC80 AUG*** |
| UB2389 | *MAT***a** *amn1::KanMX6 Ndc80-3V5:KanMX ura3::pGPD1-GAL4(848).ER::URA3* |
| UB2531 | *MAT***a** */MATalpha irt1:cup1::Hphmx/irt1:cup1::Hphmx ime4::cup1::NAT/ime4::cup1::NAT* |
| UB2936 | *MAT***a** */MATalpha ura3::pGPD1-GAL4(848).ER::URA3/ura3::pGPD1-GAL4(848).ER::URA3 GAL-NDT80::TRP1/GAL-NDT80::TRP1 leu2::pURA3-TetR-GFP::LEU2 CENV::TetOx224::HIS3 HisMX:Δ9AUG-5'UTR-Ndc80-3V5:CNAT/HisMX:Δ9AUG-5'UTR-Ndc80-3V5:CNAT pCUP-CLB3::KANMX*  ***ATG-ATC mutation in 9 of the 9 potential upstream start codons within NDC80-5'UTR. The leader sequence contains 2 other point mutations, in addition to the ATCs.*** |
| UB2940 | *MAT***a** */MATalpha ura3::pGPD1-GAL4(848).ER::URA3/ura3::pGPD1-GAL4(848).ER::URA3 GAL-NDT80::TRP1/GAL-NDT80::TRP1 leu2::pURA3-TetR-GFP::LEU2 CENV::TetOx224::HIS3 pCUP-CLB3::KANMX HIS3MX::(Δ-600 to -400)-NDC80-3V5::KanMX/HIS3MX::(Δ-600 to -400 bp)-NDC80-3V5::KanMX* |
| UB2942 | *MAT***a** */MATalpha ura3::pGPD1-GAL4(848).ER::URA3/ura3::pGPD1-GAL4(848).ER::URA3 GAL-NDT80::TRP1/GAL-NDT80::TRP1 leu2::pURA3-TetR-GFP::LEU2 CENV::TetOx224::HIS3 Ndc80-3V5:KanMX/Ndc80-3V5:KanMX* |
| UB3262 | *MAT***a** *ndc80Δ:KanMX4 leu2::NDC80-3V5:LEU2* |
| UB3301 | *MAT***a** */MATalpha UME6-3V5::His3MX/UME6-3V5::His3MX irt1:cup1::Hphmx/irt1:cup1::Hphmx ime4::cup1::NAT/ime4::cup1::NAT* |
| UB3392 | *MAT***a** */MATalpha GAL-NDT80::TRP1/GAL-NDT80::TRP1 ura3::pGPD1-GAL4(848).ER::URA3/ura3::pGPD1-GAL4(848).ER::URA3 ndc80∆(-1000 and ORF):KanMX4/ndc80∆(-1000 and ORF):KanMX4 leu2::mse-NDC80-3V5:LEU2/leu2::mse-NDC80-3V5:LEU2* |
| UB4074 | *MAT***a** */MATalpha GAL-NDT80::TRP1/GAL-NDT80::TRP1 ura3::pGPD1-GAL4(848).ER::URA3/ura3::pGPD1-GAL4(848).ER::URA3 ndc80Δ(-1000 and ORF):KanMX4/ndc80Δ(-1000 and ORF):KanMX4 leu2::NDC80-3V5:LEU2/leu2::NDC80-3V5:LEU2* |
| UB4212 | *MAT***a** *leu2::urs1Δ-NDC80-3V5:LEU2 ndc80Δ::KanMX* |
| UB4361 | *MAT***a** */MATalpha GAL-NDT80::TRP1/GAL-NDT80::TRP1 ura3::pGPD1-GAL4(848).ER::URA3/ura3::pGPD1-GAL4(848).ER::URA3 SPC24-3V5:KanMX/SPC24-3V5:KanMX NUF2-3V5:HisMX/NUF2-3V5:HisMX NDC80-3V5:CNAT/NDC80-3V5:CNAT* |
| UB4432 | *MAT***a** */MATalpha ura3::pGPD1-GAL4(848).ER::URA3/ura3::pGPD1-GAL4(848).ER::URA3 GAL-NDT80::TRP1/GAL-NDT80::TRP1 CENV::TetOx224::HIS3 leu2::pURA3-TetR-GFP::LEU2 NDC80-3V5:KanMX* |
| UB4434 | *MAT***a** */MATalpha ura3::pGPD1-GAL4(848).ER::URA3/ura3::pGPD1-GAL4(848).ER::URA3 GAL-NDT80::TRP1/GAL-NDT80::TRP1 CENV::TetOx224::HIS3 leu2::pURA3-TetR-GFP::LEU2 NDC80-3V5:KanMX pCUP-CLB3::KANMX* |
| UB4436 | *MAT***a** */MATalpha ura3::pGPD1-GAL4(848).ER::URA3/ura3::pGPD1-GAL4(848).ER::URA3 GAL-NDT80::TRP1/GAL-NDT80::TRP1 CENV::TetOx224::HIS3 leu2::pURA3-TetR-GFP::LEU2 Ndc80-3V5:KanMX pCUP-Nuf2::KANMX pCUP-CLB3::KANMX* |
| UB5101 | *MAT***a** */MATalpha pCUP-IME1::NAT/pCUP-IME1::NAT pCUP-IME4::NAT/pCUP-IME4::NAT nuf2::KanMX/nuf2::KanMX leu2::NDC80(-1000 to -1)-NUF2-3V5:LEU2/leu2::NDC80(-1000 to -1)-NUF2-3V5:LEU2* |
| UB5103 | *MAT***a** */MATalpha pCUP-IME1::NAT/pCUP-IME1::NAT pCUP-IME4::NAT/pCUP-IME4::NAT nuf2::KanMX/nuf2::KanMX leu2::NUF2-3V5:LEU2/leu2::NUF2-3V5:LEU2* |
| UB5437 | *MAT***a** */MATalpha ndc80Δ(-1000 and ORF):KanMX4/ndc80Δ(-1000 and ORF):KanMX4 leu2::mse-NDC80-3V5:LEU2/leu2::mse-NDC80-3V5:LEU2 CENV::tetOx224::HIS3/CENV::tetOx224::HIS3 his3::pURA3-TetR-GFP::HIS3/his3::pURA3-TetR-GFP::HIS3* |
| UB5473 | *MAT***a** */MATalpha leu2::urs1Δ-NDC80-3V5:LEU2/leu2::urs1Δ-NDC80-3V5:LEU2 ndc80Δ(-1000 and ORF):KanMX4/ndc80Δ(-1000 and ORF):KanMX4* |
| UB5875 | *MAT***a** */MATalpha leu2::NDC80-3V5:LEU2/leu2::NDC80-3V5:LEU2 ndc80Δ(-1000 and ORF):KanMX4/ndc80Δ(-1000 and ORF):KanMX4* |
| UB5876 | *MAT***a** */MATalpha ndc80∆(-1000 and ORF):KanMX4/ndc80∆(-1000 and ORF):KanMX4 leu2::NDC80-3V5:LEU2/leu2::NDC80-3V5:LEU2 CENV::tetOx224::HIS3/CENV::tetOx224::HIS3 his3::pURA3-TetR-GFP::HIS3/his3::pURA3-TetR-GFP::HIS3* |
| UB6075 | *MAT***a** */Matalpha irt1:cup1::Hphmx/irt1:cup1::Hphmx ime4::cup1::NAT/ime4::cup1::NAT leu2::urs1Δ-NDC80-3V5:LEU2/leu2::urs1Δ-NDC80-3V5:LEU2 ndc80∆(-1000 and ORF):KanMX4/ndc80∆(-1000 and ORF):KanMX4* |
| UB6077 | *MAT***a** */Matalpha irt1:cup1::Hphmx/irt1:cup1::Hphmx ime4::cup1::NAT/ime4::cup1::NAT leu2::(-295::ADH1)-NDC80-3V5:LEU2/leu2::(-295::ADH1)-NDC80-3V5:LEU2 ndc80Δ(-1000 and ORF):KanMX4/ndc80Δ(-1000 and ORF):KanMX4* |
| UB6079 | *MAT***a** */Matalpha irt1:cup1::Hphmx/irt1:cup1::Hphmx ime4::cup1::NAT/ime4::cup1::NAT leu2::(Δ-600 to -479)-NDC80-3V5:LEU2/leu2::(Δ-600 to -479)-NDC80-3V5:LEU2 ndc80∆(-1000 and ORF):KanMX4 /ndc80∆(-1000 and ORF):KanMX4* |
| UB6181 | *MAT***a** */Matalpha irt1:cup1::Hphmx/irt1:cup1::Hphmx ime4::cup1::NAT/ime4::cup1::NAT leu2::Δ6AUG-NDC80-3V5:LEU2/leu2::Δ6AUG-NDC80-3V5:LEU2 ndc80Δ(-1000 and ORF):KanMX4/ndc80Δ(-1000 and ORF):KanMX4*  ***ATG-ATC mutation in 6 of the 9 potential upstream start codons within NDC80-5'UTR*** |
| UB6183 | *MAT***a** */MATalpha irt1:cup1::Hphmx/irt1:cup1::Hphmx ime4::cup1::NAT/ime4::cup1::NAT leu2::Δ9AUG-NDC80-3V5:LEU2/leu2::Δ9AUG-NDC80-3V5:LEU2 ndc80Δ(-1000 and ORF):KanMX4 /ndc80Δ(-1000 and ORF):KanMX4*  ***ATG-ATC mutation in 9 of the 9 potential upstream start codons within NDC80-5'UTR*** |
| UB6190 | *MAT***a** */MATalpha irt1:cup1::Hphmx/irt1:cup1::Hphmx ime4::cup1::NAT/ime4::cup1::NAT ndc80Δ(-1000 and ORF):KanMX4 /ndc80Δ(-1000 and ORF):KanMX4 leu2::NDC80-3V5:LEU2/leu2::NDC80-3V5:LEU2* |
| UB6295 | *MAT***a** *HISMX:pGAL-Ndc80-3V5:KanMX trp1::pGAL-dCas9-MxiI::TRP1 ura3::pGPD1-GAL4(848).ER::URA3 2micron_plasmid_LEU2*  ***pGAL integrated 536 bp upstream of NDC80 AUG*** |
| UB6297 | *MAT***a** *HISMX:pGAL-Ndc80-3V5:KanMX trp1::pGAL-dCas9-MxiI::TRP1 ura3::pGPD1-GAL4(848).ER::URA3 2micron_plasmid_LEU2_NDC80guideA*  ***pGAL integrated 536 bp upstream of NDC80 AUG*** |
| UB6299 | *MAT***a** *HISMX:pGAL-Ndc80-3V5:KanMX trp1::pGAL-dCas9-MxiI::TRP1 ura3::pGPD1-GAL4(848).ER::URA3 2micron_plasmid_LEU2_NDC80guideB*  ***pGAL integrated 536 bp upstream of NDC80 AUG*** |
| UB6301 | *MAT***a** *HISMX:pGAL-Ndc80-3V5:KanMX trp1::pGAL-dCas9-MxiI::TRP1 ura3::pGPD1-GAL4(848).ER::URA3 2micron_plasmid_LEU2_NDC80guideC*  ***pGAL integrated 536 bp upstream of NDC80 AUG*** |
| UB6302 | *MAT***a** *HISMX:pGAL-Ndc80-3V5:KanMX trp1::pGAL-dCas9-MxiI::TRP1 ura3::pGPD1-GAL4(848).ER::URA3 2micron_plasmid_LEU2_NDC80guideD*  ***pGAL integrated 536 bp upstream of NDC80 AUG*** |
| UB6760 | *MAT***a** */MATalpha Ume6-3V5::His3MX/Ume6-3V5::His3MX irt1:cup1::Hphmx/irt1:cup1::Hphmx ime4::cup1::NAT/ime4::cup1::NAT leu2::urs1Δ-NDC80:LEU2/leu2::urs1Δ-NDC80:LEU2 ndc80Δ(-1000 and ORF):KanMX4/ndc80Δ(-1000 and ORF):KanMX4* |
| UB7496 | *MAT***a** */MATalpha TRP1::GAL-NDT80-3V5::KanMX/TRP1::GAL-NDT80-3V5::KanMX ura3::pGPD1-GAL4(848).ER::URA3/ura3::pGPD1-GAL4(848).ER::URA3 ndc80Δ(-1000 and ORF):KanMX4/ndc80Δ(-1000 and ORF):KanMX4 leu2::mse-NDC80:LEU2/leu2::mse-NDC80:LEU2* |
| UB7997 | *MAT***a** */MATalpha GAL-NDT80::TRP1/GAL-NDT80::TRP1 ura3::pGPD1-GAL4(848).ER::URA3/ura3::pGPD1-GAL4(848).ER::URA3 leu2::NDC80:LEU2/leu2::NDC80:LEU2 ndc80Δ(-1000 and ORF):KanMX4/ndc80Δ(-1000 and ORF):KanMX4* |
| UB7999 | *MAT***a** */MATalphaTRP1::GAL-NDT80-3V5::KanMX/TRP1::GAL-NDT80-3V5::KanMX ura3::pGPD1-GAL4(848).ER::URA3/ura3::pGPD1-GAL4(848).ER::URA3 leu2::NDC80:LEU2/leu2::NDC80:LEU2 ndc80Δ(-1000 and ORF):KanMX4/ndc80Δ(-1000 and ORF):KanMX4* |
| UB8001 | *MAT***a** *HISMX:pGAL-Ndc80-3V5:KanMX ura3::pGPD1-GAL4(848).ER::URA3 leu2::NDC80-3V5:LEU2* ***pGAL integrated 536 bp upstream of Ndc80 AUG*** |
| UB8144 | *MAT***a** */MATalpha GAL-NDT80::TRP1/GAL-NDT80::TRP1 ura3::pGPD1-GAL4(848).ER::URA3/ura3::pGPD1-GAL4(848).ER::URA3 ZIP1::GFP(700)/ZIP1::GFP(700)*  ***GFP is inserted internally of the coding region of ZIP1*** |
| UB8682 | *MAT***a** *ura3::pGPD1-GAL4(848).ER::URA3 amn1::HygB CENV::TetOx224::HIS3 leu2::pURA3-TetR-GFP::LEU2 SPC42-mCherry::NAT* |
| UB8684 | *MAT***a** *ura3::pGPD1-GAL4(848).ER::URA3 HISMX:pGAL-Ndc80-3V5:KanMX amn1::HygB CENV::TetOx224::HIS3 leu2::pURA3-TetR-GFP::LEU2 SPC42-mCherry::NAT*  ***pGAL integrated 536 bp upstream of Ndc80 AUG*** |
| UB9243 | *MAT***a** */MATalpha irt1:cup1::Hphmx/irt1:cup1::Hphmx ime4::cup1::NAT/ime4::cup1::NAT leu2::uORF(mini)-NDC80-3V5::LEU2/leu2::uORF(mini)-NDC80-3V5::LEU2 ndc80Δ(-1000 and ORF):KanMX4/ndc80Δ(-1000 and ORF):KanMX4* |
| UB12543 | *MAT****a*** */MATalpha ura3::pGPD1-GAL4(848).ER::URA3/ ura3::pGPD1-GAL4(848).ER::URA3 GALNDT80::TRP1/ GAL-NDT80::TRP1* ***CENV::TetOx224::HIS3 leu2::pURA3-TetR-GFP::LEU2*** *KANMX::pCUP-SPC24-3V5::HisMX pCUP-CLB3::KANMX* |
| UB12547 | *MAT****a*** */MATalpha ura3::pGPD1-GAL4(848).ER::URA3/ ura3::pGPD1-GAL4(848).ER::URA3 GALNDT80::TRP1/ GAL-NDT80::TRP1* ***CENV::TetOx224::HIS3 leu2::pURA3-TetR-GFP::LEU2*** *HISMX::pCUP-SPC25-3V5::KanMX pCUP-CLB3::KANMX* |
| UB12662 | *MAT****a*** */MATalpha ura3::pGPD1-GAL4(848).ER::URA3/ ura3::pGPD1-GAL4(848).ER::URA3 GAL-NDT80::TRP1/ GAL-NDT80::TRP1* ***CENV::TetOx224::HIS3 leu2::pURA3-TetR-GFP::LEU2*** *KANMX::pCUP-NUF2-3V5::HISMX pCUP-CLB3::KANMX* |
| FW1208 | *MAT***a** */MATalpha UME6-3V5::His3MX/UME6-3V5::His3MX* |
| FW1511 | *MAT***a** */MATalpha* |
| FW1871 | *MAT***a** */MATalpha ime1::pCUP-IME1::NatMX/ime1::pCUP-IME1::NatMX ime4::pCUP-IME4::NatMX/ime4::pCUP-3HA-IME4::NatMX NDC80-3V5::KanMX/NDC80-3V5::KanMX (Δ-600 to -300)-NDC80::His3MX/(Δ-600 to -300)-NDC80::His3MX* |
| FW1899 | *MAT***a** */MATalpha ime1::pCUP-IME1::NatMX/ime1::pCUP-IME1::NatMX ime4::pCUP-IME4::NatMX/ime4::pCUP-IME4::NatMX NDC80-3V5:KanMX/(Δ-600 to -300)-NDC80::His3MX* |
| FW1900 | *MAT***a** */MATalpha ime1::pCUP-IME1::NatMX/ime1::pCUP-IME1::NatMX ime4::pCUP-IME4::NatMX/ime4::pCUP-IME4::NatMX NDC80-3V5:KanMX* |
| FW1902 | *MAT***a** */MATalpha ime1::pCUP-IME1::HphMX/ime1::pCUP-IME1:: HphMX ime4::pCUP-IME4::NatMX/ime4::pCUP-IME4:: NatMX NDC80-3V5::KanMX/ NDC80-3V5::KanMX* |
| FW1923 | *MAT***a** */MATalpha ime1::pCUP-IME1::NatMX/ime1::pCUP-IME1::NatMX ime4::pCUP-IME4::NatMX/ime4::pCUP-IME4::NatMX HisMX::(Δ-600 to -300)-NDC80-3V5:KanMX/NDC80* |
| FW3058 | *MAT***a** */MATalpha ime1::His3MX/ime1::His3MX ime4::pCUP-IME4::NatMX/ime4::pCUP-IME4::NatMX NDC80-3V5::KanMX/NDC80-3V5::KanMX* |

**Supplemental file 1B. Primers used for strain construction in this study.**

| **Construct name** | **Forward primer** | **Reverse primer** |
| --- | --- | --- |
| *NUF2-3V5* | TTAATAAATACATGAATGAAATGCTCGAATATATGCAAgcggccgctctagaactagtgg | CACAGAAGGGGGAGTAAAAATAAGTATACCGCTGCTAccccctcgaggtcgacggtatcg |
| *SPC24-3V5* | TCTATAAGACCAAATACATCTGGGAAAGATTAGGAAAGgcggccgctctagaactagtgg | TGCCACTGTAGTATTTTATTAATGATCTCAATTTTCAccccctcgaggtcgacggtatcg |
| *SPC25-3V5* | TTTTAGTCGTGGCCCGCGATATGCTTCTGGCATCTTTAgcggccgctctagaactagtgg | CCCAGAATAAACTGAACAGATGCGTATAAAGGCGTTAccccctcgaggtcgacggtatcg |
| *pCUP-NUF2* | TGGTAAAAAGCATGTACTGAGGAGAAAGGCTCCAGCATCCGAATTCGAGCTCGTTTAAAC | GTAGATCCAAAATGGGGAACACATCTTGATTCCTACTCATTTTATGTGATGATTGATTGATTGATTG |
| *pCUP-SPC24* | AAAGAAGTAACGAGGGAAGAGCAAGTGTAAAGGGAAGGAAGAATTCGAGCTCGTTTAAAC | ATTCAACCGGATTGTCGAGTAGGTTATCCTTTTGTGACATTTTATGTGATGATTGATTGATTGATTG |
| *pCUP-SPC25* | GATTCAATTAAAACCGCTCATACGTATACAACACATACACACATACAAGAgaattcgagctcgtttaaac | AACCCGTCCATCCGGCGCTCAAGGTCCGAAAATGCGTCTATGCTGGCCATtttatgtgatgattgattgattgattg |
| *ndc80∆(-1000 and ORF)* | AAGCAATTTGATGTCGTCTGGCAAAGTTCGAGGATAGTTAcggatccccgggttaattaa | TTGCTGTAGATTGCTCGGGTATTATATATCATTTATTTTAgaattcgagctcgtttaaac |
| *ndc80∆* | GGAGAGGTAGAATCGTCCCTG | GAGTGAGGGTGGAATTGAAC |
| *nuf2∆* | CGTATTGGGTTTATCGCTTTGG | CATCTTGTGTGACTTGCC |
| *pGAL-NDC80^luti^* | GATATCTGTTCAGCCAACATTATAAAAAAGATGGGCGCTT GAATTCGAGCTCGTTTAAAC | CTGATGTAAGTTAATTAGAAGGAGTGATATCTGTAAGAGTATTTTGAGATCCGGGTTTT |
| *(∆-600 to -300)* | AAAGTAACATTTACCCGGATATCTGTTCAGCCAACATTAT  CGGATCCCCGGGTTAATTAA | TATTTTAACCGCTAATCGCAATAGACTGCTTACATCTTTAGAATTCGAGCTCGTTTAAAC |

| **LEU2 single integration plasmids** | **Plasmid number** |
| --- | --- |
| *NDC80* (untagged) | pUB872 |
| *NDC80* | pUB873 |
| *∆9AUG* | pUB880 |
| *∆6AUG* | pUB895 |
| *mini uORF* | pUB905 |
| *∆NDC80^luti^ (∆-600 to -479)* | pUB883 |
| *NDC80^luti^-NUF2* | pUB665 |
| *NUF2* | pUB666 |
| *ndc80-∆urs1* | pUB882 |
| *ndc80-mse* | pUB875 |
| *NDC80^luti^-Ter* | pUB894 |

**Supplemental file 1C. Plasmids used for strain construction in this study.**

| **2-micron plasmids** | **Guide RNA template** | **Plasmid number** |
| --- | --- | --- |
| Guide A | AGCAACAATTCGCCACAAGA | pUB759 |
| Guide B | GCCACAAGAAGGTCTCTGTA | pUB760 |
| Guide C | TAGGAGCAGGGCTCTTGTCA | pUB761 |
| Guide D | CTGTGAATGCAAAGGAAAGA | pUB762 |
| Empty vector | N/A | pUB52 |

**Supplemental file 1D. Primers used for quantitative PCR and northern blotting in this study.**

| **Primer Name** | **Oligonucleotide sequence from 5’ to 3’** |
| --- | --- |
| *NDC80_probe_F* | GGAGAGGTAGAATCGTCCCTG |
| *NDC80_probe_R* | CTCCTCTTGAATAGCGCTTTGG |
| *NUF2_probe_F* | AACAGGGGATGGTCACTTACAGG |
| *NUF2_probe_R* | CCCACAAGTTCCGTTTCAGTTCG |
| *SCR1_probe_F* | GAAGTGTCCCGGCTATAATAAA |
| *SCR1_probe_R* | GACGCTGGATAAAACTCCCC |
| *CIT1_probe_F* | CCGTGTTAGACCCCGAAGAAG |
| *CIT1_probe_R* | GGGCAGAAACGTTACCACCTTC |
| *NDC80_2_F* | ACCCGGATATCTGTTCAGCC |
| *NDC80_2_R* | TGTGGCGAATTGTTGCTCTT |
| *NDC80_6_F* | GGTTGAGAGCCCCGTTAAGT |
| *NDC80_6_R* | TTGGCACTTTCAGTATGGGT |
| *NDC80_7_F* | CCCATACTGAAAGTGCCAAAAGA |
| *NDC80_7_R* | GGGACGATTCTACCTCTCCTGTG |
| *pNUF2_F* | GTCGCTGCGTATTCAGCGTA |
| *pNUF2_R* | GAACGCTGATATACTCGACTAAC |
| *ACT1_F* | GTACCACCATGTTCCCAGGTATT |
| *ACT1_R* | AGATGGACCACTTTCGTCGT |
| *NDC80_9_F* | TGCAAAGCTCAACAAGTACTGA |
| *NDC80_9_R* | TGCAGTTGGTATTTGGGACG |
| *NDC80_ORF_F* | ATCCGAGTGTGAACTGAAAGAAG |
| *NDC80_ORF_R* | GAACTGCTCAGTTGAAATTCCC |
| *IME2_URS1_F* | CCAAATACGCTTTTTAAACTTGG |
| *IME2_URS1_R* | CTCAAATAGCCGCCGTAAC |
| *MAM1_MSE_F* | CACAATTGAAATCCGAGCTGT |
| *MAM1_MSE_R* | CATCTGAATTTTGAATGGCTTT |

**Supplemental file 1E. smFISH oligonucleotide probes used in this study.**

The NDC80ORF (Q 670) probe set consists of a mixture of thirty 20-mer oligonucleotide probes that tile the common region shared between *NDC80^luti^* and *NDC80^ORF^*. Each individual probe is labeled with the Quasar 670® dye.

The NDC80Long (CF 590) probe set consists of a mixture of twenty 20-mer oligonucleotide probes that tile the unique 5’ region of *NDC80^luti^*. Each individual probe is labeled with the CAL Fluor Red 590® dye.

| **Probe name** | **Probe sequence** | **Probe name** | **Probe sequence** |
| --- | --- | --- | --- |
| NDC80ORF_1 | tccatgtgatgtagcacatg | NDC80Long_1 | ttttgctttcttactgatgt |
| NDC80ORF_2 | cagttggtatttgggacgta | NDC80Long_2 | tcttgtggcgaattgttgct |
| NDC80ORF_3 | ctgtttcttctcctcaattg | NDC80Long_3 | cctattgaccctacagagac |
| NDC80ORF_4 | atgtcggttagaccttgatt | NDC80Long_4 | tttccctgacaagagccctg |
| NDC80ORF_5 | ttgtattcctggcaatactc | NDC80Long_5 | tctttcctttgcattcacag |
| NDC80ORF_6 | ttttatttatgcctcctgtg | NDC80Long_6 | aatgcttttcggacctccaa |
| NDC80ORF_7 | aatgctgtaccatttgtacc | NDC80Long_7 | tctcttcaatcctaacatca |
| NDC80ORF_8 | tgacgctgtttctactgttg | NDC80Long_8 | ctggcacatagtacggtgaa |
| NDC80ORF_9 | gctgccaagttgatttattg | NDC80Long_9 | ttcaatgttcagttataacc |
| NDC80ORF_10 | agtttttgtctcttagtggc | NDC80Long_10 | cagcccataatcacgatatt |
| NDC80ORF_11 | aatctcctcttgaatagcgc | NDC80Long_11 | aatacttaacggggctctca |
| NDC80ORF_12 | tagtaaagccgtaacctgga | NDC80Long_12 | atagactgcttacatcttta |
| NDC80ORF_13 | acctacagccgaaatttgtg | NDC80Long_13 | ggtattttaaccgctaatcg |
| NDC80ORF_14 | atgccaagaaatttgtgcca | NDC80Long_14 | cagtatgggtaacccttgaa |
| NDC80ORF_15 | gctggctcagaattgttatt | NDC80Long_15 | tctttttttcttttggcact |
| NDC80ORF_16 | ctgttcgtccaaagtcttta | NDC80Long_16 | gactatatcattccatacgt |
| NDC80ORF_17 | ttcagttcttgcatcgaagg | NDC80Long_17 | ttttaggaaatattagtttt |
| NDC80ORF_18 | tggttttgatcttttggctt | NDC80Long_18 | ccatttttggtgttgtttgt |
| NDC80ORF_19 | ctcagttgaaattccctttt | NDC80Long_19 | gtgaatgtattccaattatt |
| NDC80ORF_20 | tttatcaagttccctagtca | NDC80Long_20 | cagggacgattctacctctc |
| NDC80ORF_21 | ttccagctttctggatttaa |  |  |
| NDC80ORF_22 | cgaatcgtattgcctcaacg |  |  |
| NDC80ORF_23 | gactcgttaattccagatcc |  |  |
| NDC80ORF_24 | gttgtctttctcaatggttt |  |  |
| NDC80ORF_25 | ttcgttctcttgcttagaga |  |  |
| NDC80ORF_26 | ctcaattctttgcgctacta |  |  |
| NDC80ORF_27 | gaagttaccaattcctcagc |  |  |
| NDC80ORF_28 | ttcttccaattttagttccg |  |  |
| NDC80ORF_29 | cttgtatcgtttcctgttta |  |  |
| NDC80ORF_30 | catgtatcacttgttggtgt |  |  |

**Supplemental file 1E (continued). smFISH oligonucleotide probes used in this study.**

The NDC80Odd (CF 590) probe set consists of a mixture of twenty-seven 20-mer oligonucleotide probes that tile *NDC80^ORF^*. Each individual probe is labeled with the CAL Fluor Red 590® dye.

The NDC80Even (Q 670) probe set consists of a mixture of twenty-seven 20-mer oligonucleotide probes that tile *NDC80^ORF^*. Each individual probe is labeled with the Quasar 670® dye.

| **Probe name** | **Probe sequence** | **Probe name** | **Probe sequence** |
| --- | --- | --- | --- |
| NDC80Odd_1 | atttttcttgttccgtttca | NDC80Even_1 | atagtacaccctaacgttta |
| NDC80Odd_2 | acgggtatctcttatggaat | NDC80Even_2 | acttgttgagctttgcattt |
| NDC80Odd_3 | tgtgatgtagcacatgttga | NDC80Even_3 | gacgtaaaccgatgagggtc |
| NDC80Odd_4 | acgatgttgcagttggtatt | NDC80Even_4 | ctgtttcttctcctcaattg |
| NDC80Odd_5 | atgtcggttagaccttgatt | NDC80Even_5 | ttgtattcctggcaatactc |
| NDC80Odd_6 | ttttatttatgcctcctgtg | NDC80Even_6 | aatgctgtaccatttgtacc |
| NDC80Odd_7 | tgacgctgtttctactgttg | NDC80Even_7 | gctgccaagttgatttattg |
| NDC80Odd_8 | ctattgctcagatgttgctg | NDC80Even_8 | agtttttgtctcttagtggc |
| NDC80Odd_9 | aatctcctcttgaatagcgc | NDC80Even_9 | aataaaccccttttgagtgg |
| NDC80Odd_10 | tagtaaagccgtaacctgga | NDC80Even_10 | acctacagccgaaatttgtg |
| NDC80Odd_11 | atgccaagaaatttgtgcca | NDC80Even_11 | tttgttcgtaccatccaatg |
| NDC80Odd_12 | ttcaagcacatatccagttt | NDC80Even_12 | gctggctcagaattgttatt |
| NDC80Odd_13 | ctgttcgtccaaagtcttta | NDC80Even_13 | gctcatatctttcttgtctt |
| NDC80Odd_14 | attaacagtttctccaccat | NDC80Even_14 | ttcagttcttgcatcgaagg |
| NDC80Odd_15 | gattgtcattttgggtttgt | NDC80Even_15 | tggttttgatcttttggctt |
| NDC80Odd_16 | cttttcaaagccttccattt | NDC80Even_16 | ttcttgactcttttgcttca |
| NDC80Odd_17 | ggatttcatcttttccagtt | NDC80Even_17 | tcttcttctttcagttcaca |
| NDC80Odd_18 | ctcagttgaaattccctttt | NDC80Even_18 | tttctctttcttggttttgt |
| NDC80Odd_19 | tttatcaagttccctagtca | NDC80Even_19 | ttccagctttctggatttaa |
| NDC80Odd_20 | agcttttgaatattccctcg | NDC80Even_20 | cgaatcgtattgcctcaacg |
| NDC80Odd_21 | ccacgcgatctggttaaatt | NDC80Even_21 | ctcgtaggagatagcttcat |
| NDC80Odd_22 | gactcgttaattccagatcc | NDC80Even_22 | gttgtctttctcaatggttt |
| NDC80Odd_23 | attcgcttcggataattcca | NDC80Even_23 | ttcgttctcttgcttagaga |
| NDC80Odd_24 | ctcaattctttgcgctacta | NDC80Even_24 | gaagttaccaattcctcagc |
| NDC80Odd_25 | ttcttccaattttagttccg | NDC80Even_25 | cttgtatcgtttcctgttta |
| NDC80Odd_26 | catgtatcacttgttggtgt | NDC80Even_26 | ttcgttttcagagttttcca |
| NDC80Odd_27 | actcttcaatgacgtttcct | NDC80Even_27 | atgttcagtttcaaactcca |

**Supplemental file 1F. Summarized smFISH results for this study.**

| **Figure** | **Conditions** | **Median of the number of *NDC80^luti^* transcripts per cell** | **Median of the number of *NDC80^ORF^* transcripts per cell** |
| --- | --- | --- | --- |
| 2D | Vegetative growth | 0 | 4 |
|  | Meiotic prophase | 21 | 3 |
|  | Meiosis I | 6 | 24 |

Note: This strain (UB8144) harbored the *pGAL-NDT80* *GAL4-ER* system. Meiotic prophase cells were defined as Zip1-GFP positive cells, and meiosis I cells were defined as Zip1-GFP negative cells present after release from the *pGAL-NDT80* block.

| **Figure** | **Conditions** | **Median of the number of *NDC80^luti^* transcripts per cell** | **Median of the number of *NDC80^ORF^* transcripts per cell** |
| --- | --- | --- | --- |
| Fig. 2-S6 | wild-type  Pre-meiotic stage | 0 | 5 |
| Fig. 2-S6, 4C, 6H | wild-type  Meiotic prophase | 15 | 4 |
| 4C | *∆NDC80^luti^*  Meiotic prophase | 1 | 6 |
| 6H | *ndc80-urs1∆*  Meiotic prophase | 5 | 4 |

Note: Different from in the strain UB8144 listed above, these 3 strains (wild-type cells (UB6190), ∆*NDC80^luti^* (UB6079), and *ndc80-urs1∆* (UB6075)) harbored the *pCUP-IME1 pCUP-IME4* system. Meiotic prophase was staged as 2 hr after Cu addition, and pre-meiotic cells was staged as prior to Cu addition.

| **Figure** | **Conditions** | **Median of the number of *NDC80^luti^* transcripts per cell** | **Median of the number of *NDC80^ORF^* transcripts per cell** |
| --- | --- | --- | --- |
| 6E | wild-type  Vegetative growth | 0 | 3 |
|  | *ndc80-urs1∆*  Vegetative growth | 3 | 2 |

Note: These two strains (wild-type cells (UB5875) and *ndc80-urs1∆* (UB5473)) do not carry either the *pGAL-NDT80 GAL4-ER* or the *pCUP-IME1* *pCUP-IME4* system.

**Supplemental file 1G. Predicted peptide sequences for the putative AUG uORFs.**

| Species | Number of uORFs | Predicted uORF peptide sequence |
| --- | --- | --- |
| *S. bayanus* | 10 | MARRTEQDNKKKVTRQTNAG*  MRADLMPFDSLW*  MVMVFKYRKEHQRCKYVSRFVGTRWLRPVLT*  MVFKYRKEHQRCKYVSRFVGTRWLRPVLT*  MCLDLSVHGG*  MI*  MSDHGGNKHTARSGWLCSAVKGLSMALAT*  MVVINIQLDRVGSARRLRGFQWHLLPNLLPSAAKLRYPASNTQKDAFLRTK*  MIIHKTKISKINKRHQKWQKTKEIDSIEEVGIYHWLFRDRTVKK*  MAKNKRNR* |
| *S. kudriavzevii* | 8 | MRLVLE*  MRTKRLRVLNQFRFIGAKIEQRLCCSAC*  MLEPIAIVL*  MS*  MALSDS*  MGYPSQEIQFNASPRTK*  MIYPQNENTQNKQTTPKMVKQKR*  MVKQKR* |
| *S. mikatae* | 5 | MIQVLLVYYHQIVVRE*  MLRKAKKTGGPKSIGVRIGEELYYTTGHI*  MTTSRWDPCLVTLNI*  MFSKVNKEHQK*  MEIDNLGGG* |
| *S. paradoxus* | 6 | MS*  MHIGWEPC*  MLTMVRM*  MDHNV*  MIYTKLNSPKINKQHQKWQELLE*  MARIIGIDCVGEVQSVPSFCQKLKQSKKNN* |
| *S. cerevisiae* | 9 | MQRKDGWRSEKHY*  MLGLKRNSPYYVPDGYN*  MCQMVITEH*  MVITEH*  MGWLRAPLSIVKDVSSLLRLAVKIPFQGLPILKVPKEKKITYGMI*  M*  ME*  MI*  MVEIIGIHSQER* |
